# Evalution of the “Iceberg Phenomenon” in Johne's Disease through Mathematical Modelling

**DOI:** 10.1371/journal.pone.0076636

**Published:** 2013-10-22

**Authors:** Gesgam Magombedze, Calistus N. Ngonghala, Cristina Lanzas

**Affiliations:** 1 National Institute for Mathematical and Biological Synthesis (NIMBioS), University of Tennessee, Knoxville, Tennessee, United States of America; 2 Department of Biomedical and Diagnostic Sciences, College of Veterinary Medicine University of Tennessee, Knoxville, Tennessee, United States of America; University of Illinois at Urbana-Champaign, United States of America

## Abstract

Johne's disease (JD) is a chronic, enteric disease in ruminants caused by *Mycobacterium avium* subsp. *paratuberculosis* (MAP). Disease progression follows four distinct stages: silent, subclinical, clinical and advanced. Available diagnostic tests have poor sensitivity and cannot detect early stages of the infection; as a result, only animals in the clinical and advanced stages, which represent the tip of the ‘iceberg’, are identified through testing. The Iceberg Phenomenon is then applied to provide estimates for JD prevalence. For one animal in the advanced stage, it is assumed that there are one to two in the clinical stage, four to eight in the subclinical stage, and ten to fourteen in the silent stage. These ratios, however, are based on little evidence. To evaluate the ratios, we developed a deterministic ordinary differential equation model of JD transmission and disease progression dynamics. When duration periods associated with the natural course of the disease progression are used, the above ratios do not hold. The ratios used to estimate JD prevalence need to be further investigated.

## Introduction

*Mycobacterium avium* subsp. *paratuberculosis* (MAP) is the etiological agent of Johne's disease (JD) in ruminants [1]. JD is a chronic, contagious granulomatous enteritis characterized by persistent and progressive diarrhea, weight loss, debilitation, and death. JD is a costly disease in dairy farming because it causes reduced milk production, increased cattle mortality and premature culling of sick cattle, and reduced sale price for cattle from regions with high disease prevalence [2]. In addition, MAP may be a zoonotic hazard, as it has been implicated as a probable cause of human Crohn's disease [3], [4].

The primary route of transmission of JD is fecal-oral. MAP exposure sources include contaminated teats of adult cattle during suckling, milk or colostrum containing MAP bacilli, or contaminated pasture, feed, soil, water, and other surfaces [1], [5], [6]. MAP can also be transmitted directly from cattle to cattle through infected semen when herds share a bull for breeding [6]. Utero transmission is highly probable from dam to calves [7]. Neonates are more susceptible than adults because of their undeveloped immune system [5]. Although, ingestion of bacilli by adult cattle does not necessarily lead to infection; repeated uptake of high doses of bacilli results in adult cattle infection [1], [5], [8]–[10]. After infection, disease progression follows four distinct stages: (i) silent or latent, (ii) subclinical, (iii) clinical and (iv) advanced [1], [6], [10], [11]. Infected cattle begin shedding bacilli after an unpredictable but lengthy latent period, which ranges from 2 to 10 years, and shedding increases with disease progression. In the subclinical stage, animals shed minimal amounts of MAP bacteria, thereby contributing a steady stealthy contamination to the environment [5].

Currently, no cost-effective treatment is available for JD, and certification and control programs implemented in several countries have had limited success [2], [10], [12]. Therefore, there is need to invest in efforts geared at designing and implementing effective preventive and control strategies. Practical methods for early infection diagnosis are still to be developed, yet shedding is alleged to start during the subclinical stage [13], [14]. By the time an animal is diagnosed, it has long been transmitting the disease, and the environment is already contaminated. When an infected animal is finally detected in a herd, it is often a reflection of transmission events that occurred many years before, perhaps during a period as long as the subclinical duration. Thus, detection of clinically infected animals or noticeable signs of JD are just the tip of the “iceberg”. This is known as the “iceberg phenomenon” [11], [15], [16], a common phenomenon in many endemic diseases, in which more infections are unnoticed because they reside within the subclinical group or are not detected. Diagnosed clinical cases (located at the tip of the iceberg) are the first to be noticed, and subclinical cases (unobserved beneath the ocean surface) are often unnoticeable but present. In JD, the following ratios have been used to estimate the total disease burden: for one animal in the advanced stage, there are one to two animals in the clinical stage, four to eight in the subclinical stage and ten to fourteen in the silent stage [5], [11], [15]. Because of limited diagnostic and detection procedures, these ratios are loosely used to provide an estimation of true disease prevalence in a herd.

Several studies have been carried out to estimate herd-level prevalence of JD in farms and to determine the impact of culling and farm-level hygiene in controlling the disease [2], [17]–[22]. In retrospect, there is potential to underestimate the extent of contamination that can be caused by animals while in the subclinical stage, the impact this has on disease prevalence and persistence, and the corresponding effort that should be put forth to control the disease from spreading. Current control methods emphasise culling of clinical cases known to be high shedders without taking cognisance of the threat posed by subclinical cases. Therefore, predicting ratios of animals in the respective stages of the disease paints a better understanding of JD prevalence and the necessary control measures that should be put forth. In this study we developed a mathematical model to evaluate the accuracy and reliability of the ratios commonly used to describe the iceberg phenomenon in JD. For that purpose, we simulated the disease dynamics in cattle populations and evaluated the sensitivity of the predicted ratios to disease and demographic parameters. This inquiry is motivated by the lack of supporting evidence from the study that originally reported the ratios commonly used to describe the iceberg phenomenon for JD in cattle [11]. Subsequent studies have echoed and referenced this first publication [5], [6], [15], [16].

## Methods

We developed a deterministic ordinary differential equation (ODE) model to investigate the iceberg phenomenon in JD. The model apportions cattle into four classes depicting disease status: susceptible (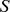), exposed or silent (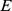), subclinical (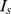), and clinical (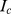). Cattle in the silent stage do not shed bacteria in their feces, subclinical cattle are low shedders, and clinical cattle are high shedders. In this study we do not regard the advanced disease stage because farmers usually cull cattle that reach this stage. Disregarding this class does not affect the scope of the study since we are interested in the contributions of the subclinical and clinical stages to disease transmission.

### JD transmission model

At any time (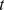), the total cattle population (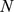) is given by 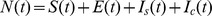. See Table 1 for a brief description of the various classes. We assume deaths occur in each of the four classes at a constant per capita rate 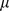. The susceptible cattle class is populated by new births from all the four classes and incoming cattle from other farms at rate 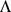, and the model does not assume vertical transmission. Cattle in this class become infected through contaminated environments at rate 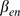, or contaminated parent cattle teats at rate (direct contact) 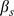 or 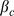 depending on whether the parent cattle is at the subclinical or clinical stage. The parameters 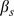 and 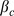 are also loosely used to capture all other possible cattle-to-cattle interactions that may result in infection transmission [17], [20]. Transmission of infection is therefore modelled by 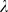 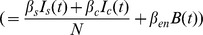, the force of infection. Upon infection, the cattle becomes exposed (enter the silent stage) to the disease and eventually progresses to the subclinical stage at rate 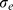. Cattle within the subclinical class progress to the clinical class at rate 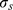 after a certain time frame. Clinical cattle also attain advanced disease stage at rate 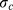 (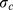 may as well be interpreted as isolation or culling of cattle progressing to the advanced stage). See Table 2 for brief descriptions of the parameters used in the model. Subclinical and clinical cattle contaminate the environment through shedding that occur at rates 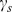 and 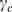, respectively, where 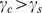, while bacilli in the contaminated environment decays at rate 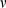. This parameter can also be explained as environmental cleaning. If there is no cleaning exercise at the farm, then 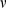 models the life span of bacilli in the environment. With cleaning, it takes a value less than the decay rate of bacilli in the environment and corresponds to how often the cleaning exercise is carried out per year.

**Table 1:** Brief description of model variables.

Variable	Epidemiological description
*S*	Susceptible cattle. Cattle that have not yet picked up MAP bacteria
*E*	Exposed cattle (silent stage). Cattle with MAP but which cannot shed the bacilli to the environment or transmit infection to other cattle
*I_s_*	Subclinical cattle. Asymptomatic low-shedding but infectious cattle
*I*_c_	Clinical cattle. Asymptomatic and high-shedding cattle
*N*	Total cattle population
*B*	Environmental contamination through shedding from subclinical and clinical cattle

**Table 2:** Parameters used in the simulations.

Parameter	Description	Range	Value used	Reference
Demographic				
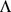	Birth and recruitment	50–100	100	Assumed
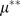	Natural death rate	0.05–0.04	0.05	–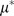	Farm animal removal rate	0.2–0.25	0.2	–
Transmission				
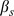	Subclinical cattle transmission rate	0.0–3.0	0.05	[17], [19], [20]
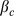	Clinical cattle transmission rate	0.0–3.0	0.1	[17], [19], [20]
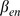	Environment transmission rate	0.0–3.0	0.025	[17], [19], [20]
Stage duration				
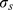	Subclinical stage duration (2–10 yrs)	0.5–0.1	0.33	[1], [11]
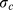	Clinical stage duration (2–4 yrs)	0.5–0.25	0.25	[1], [11]
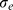	Silent stage duration (0–0.33 yrs)	0–3.5	3	[1], [11]
Environment-related				
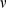	Bacilli decay rate (0.8–1.5 yrs)	0.5–1.25	0.667	[1], [27]
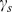	Probability of contamination by 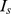 cattle	0.0–1.0	0.05	Assumed
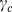	Probability of contamination by 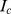 cattle	0.0–1.0	0.1	Assumed

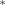
 indicates that the life span of animals on a farm is assumed to be 5 years and 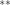 indicates that the natural life span of cattle is about 20 years. In a diary farm setting, farmers tend to keep cattle for 4 to 5 years when they are highly productive, and replace them when they age and become less productive.

**Figure 1:**
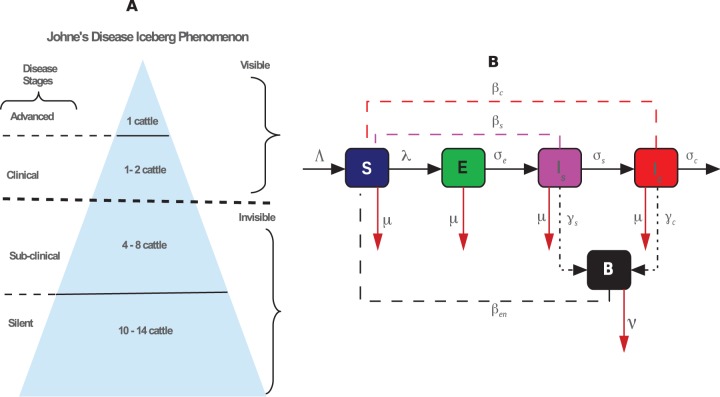
JD iceberg and transmission. A) Ratios used to describe the JD iceberg phenomenon. Infections underneath are not detectable while those that are above are visible. However, the extent of the depth of disease beneath is difficult to predict. B) Conceptual framework illustrating interactions between cattle and the environment, and the flow of cattle between the susceptible, exposed (silent), subclinical, and clinical compartments. Solid black lines represent the movement of cattle between classes. Dashed magenta, red, and black lines represent interactions between the subclinical, clinical, and the environment with susceptible cattle, respectively 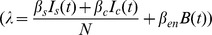. Dotted black arrows represent MAP bacteria shedding by subclinical and clinical infected cattle, while red solid lines denote cattle deaths and clearance of environmental contamination.

Using the flowchart in Figure 1B, the variables in Table 1 and the above description, we derived the following system of equations governing the dynamics of the cattle population in the presence of JD:
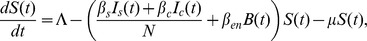


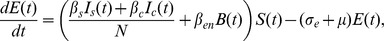


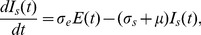
(1)

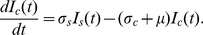



Adding the equations of the system (1) leads to the following equation that characterizes the behavior of the total cattle population:
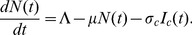
(2)See file S1 in Supporting Information S1 for positivity, well-posedness and boundedness analysis of the solutions to the system (1).

The rate of change of environmental contamination is given by the equation
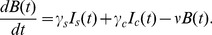
(3)


#### Model reproduction number

In this study, 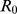 is defined as the average number of secondary infection cases that one susceptible cattle will introduce in a purely susceptible cattle population or environment for the length of time the cattle is infectious or from the time the environment is contaminated and is a reservoir for MAP bacteria. This threshold measure is used to establish conditions for the existence and stability of equilibrium solutions to the model. The basic reproduction number of the model (1) and (3) was computed using the next generation operator approach [23] to be
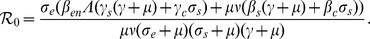



To this effect, equations (1) and (3) have a stable disease-free equilibrium solution 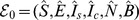 
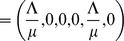
 when 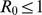 and a unique stable endemic equilibrium solution 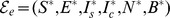 when 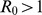. See file S1 in Supporting Information S1 for details on the computation of 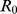, the equilibrium solutions of the model, and the local and global stability analysis of the disease-free equilibrium.

### Prevalence data and model fitting

Data was obtained from a study [22] that reports prevalence for 36 dairy herds that completed 4 or more years of testing in an Australian JD control program. Prevalence was markedly increased from 1992 to 1994 before commencement of a control program. The implemented control program involved identifying and culling animals that tested positive to the ELISA blood test. These animals were categorised as clinically infected. The developed model was fitted to the prevalence data for the time period from 1992 to 1994 before a control program was introduced. Fitting was done as a way to estimate parameters that could be used to further investigate and understand the iceberg phenomenon (see results). The least squares method was used with the *lsqcurvefit* nonlinear curve fitting function in Matlab. The model predicted prevalence,
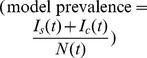
, was fitted to the prevalence reported in the study [22]. To identify parameters to fit the model, the FME inverse modelling package was used to determine a parameter set with the least collinearity index 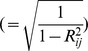, were 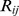 is the correlation between two sets of parameters 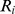 with other parameters 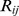 to avoid over fitting [24]. Collinearity (multicollinearity) occurs when independent variables are so highly correlated that it becomes difficult or impossible to distinguish their individual influences on the response variable.

### Model simulations

Matlab solver ode45 was used to solve the model with parameters drawn from the JD literature (Table 2). At first, model parameters obtained from literature were used in simulating the JD dynamics. However, because of uncertanity in the estimation of transmission rates, we estimated the 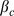 and 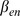 transmission rates using data from the study [22] and further used them to simulate JD transmission dynamics as a basis for making a comparison between the different sets of parameters. Our simulations were designed to investigate conditions under which the iceberg phenomenon is or is not observable using duration periods that describe the natural course of the disease [1], [10]. The animals were assumed to have either a life span of 5 years (e.g. dairy farm setting) or 20 years (natural cattle life span, e.g. beef cattle).

## Results

### Disease dynamics with naturally observed incubation periods: The ratios differ from those previously reported

**Figure 2:**
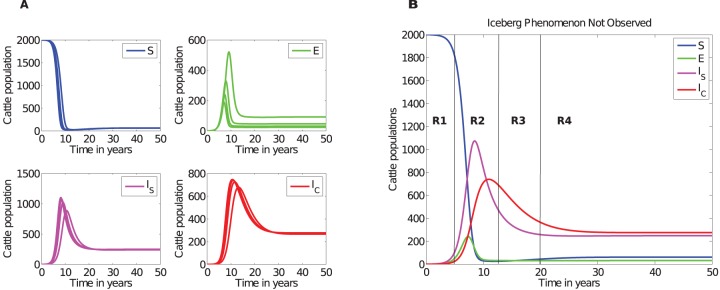
Simulated JD dynamics. A), An illustration of MAP transmission in a farm when incubation, 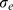, for the silent stage is varied from 4 months to 12 months in steps of 2 months. Qualitatively similar simulations are achieved with different combinations of parameters as long as 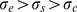. At any given time 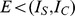. B) Comparative analysis of cattle population at different disease stages in different time regions (*see* Table 3 for ratios in different regions). Simulations were carried out using parameters given in Table 2 with 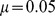 (cattle natural death rate).

Using simulated results shown in Figure 2, we calculated the ratios of cattle between the silent, subclinical and clinical stages to determine if the model could generate ratios reported in the study [11]. These ratios are given by R1, R2, R3 and R4 calculated at different time regions during the course of the disease. We investigated the ratios for two different death rates (i) 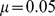, and (ii) 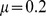, which correspond to an average cattle life span of 20 years and 5 years, respectively. See Figures 2 and file S2 in Supporting Information S1 for simulations associated with 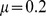, for illustrations of results obtained.

**Figure 3:**
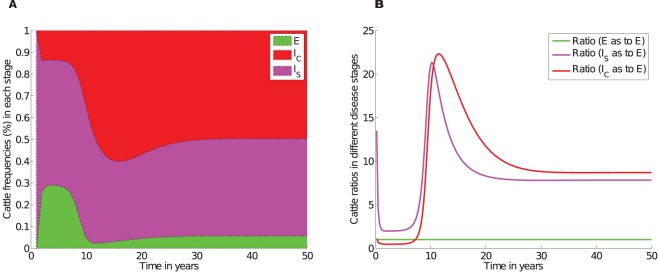
Percentages and ratios of animals in each sub-class. A) Frequency (or percentage) compositions of cattle within the exposed, subclinical, and clinical classes over the course of the disease. B) Simulated exposed, subclinical and clinical cattle ratios over the course of the disease. Ratios in each category were calculated relative to cattle in the exposed class. Parameters used are given in Table 2.

Figures 2 and 3, and Table 3 illustrate that the ratios depend on how long the disease has been established in the farm. The ratios previously suggested are not supported by our simulations. Model simulations show that at any given time, there will be more cattle in the subclinical class than in the silent class and that the number of cattle in the silent stage is marginally greater or equal to the number of cattle in the clinical stage only in the early stages (within about the first 7 years) of the disease, see Figures 2 and 3. However, it is evident that with disease progression, cattle numbers in the subclinical and clinical stages will become comparatively equal, but distinctly greater than the number of cattle in the exposed class.

**Table 3:** Approximate ratios for animal populations in different stages of JD under different time regions.

Stage	R1	R2	R3	R4
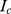 (Clinical)	30.0	600.0	500.0	275.0
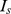 (Subclinical)	100.0	1050.0	300.0	250.0
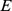 (Silent or exposed)	50.0	200.0	50.0	50.0
Ratio ( 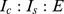 )	1:4:1	3:5:1	10:6:1	5:5:1

### Conditions for iceberg phenomenon to be observed

**Figure 4:**
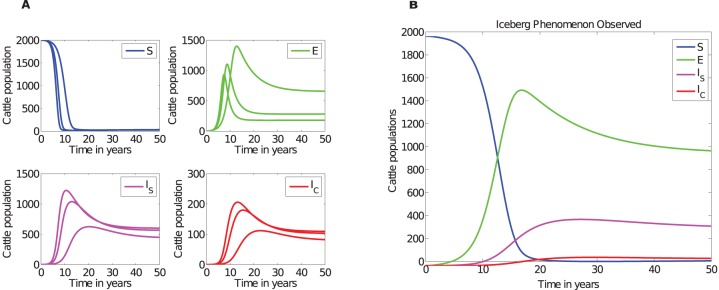
Conditions for the iceberg. Simulations demonstrating the iceberg phenomenon when 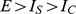 or 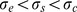 for a cattle life span of 20 years. Whenever the above inequality fails, the Iceberg phenomenon is not observable. In A, 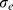 was varied between 0.02 and 0.05 in steps of 0.01. Simulations were generated using the parameters 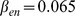, 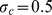, 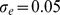, 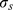, and the rest as given in Table 2.

The iceberg phenomenon as previous described is demonstrated in Figure 4, after modifying the duration periods. That is, after assuming that animals will spent more time in the silent stage than time spent in the subclinical stage before proceeding to the clinical stage. The only short-coming of this assumption, is that the subclinical stage is known to have a duration period that spans from 2 to 10 years [1], [6]. This implies that the silent stage should take a duration period longer than the range of the subclinical duration period to maintain the ratio of 4 to 8 animals in the subclinical stage to 10 to 14 animals in the silent stage. However, several studies have shown that some animals begin shedding within a period less than one year and even a short time period when there is high bacilli inoculation at infection [14], [21], [25], [26].

### Estimated parameters by fitting prevalence

**Figure 5:**
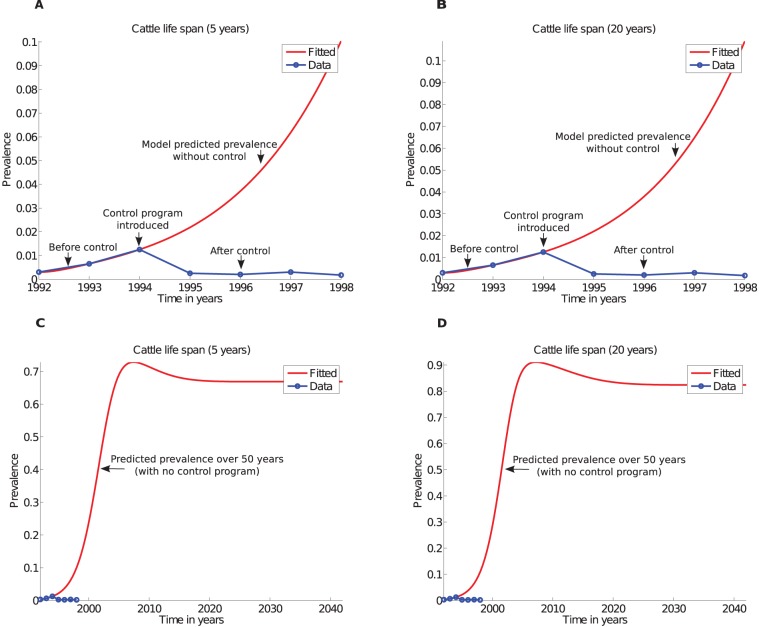
Fitted and predicted prevalence. Fitted prevalence assuming A) cattle life span of five years (farm setting), and B) cattle life span of 20 years (average cattle natural life span). C) and D) Predict disease prevalence over 50 years in the absence of control programs for cattle life spans of 5 and 20 years, respectively.

**Figure 6:**
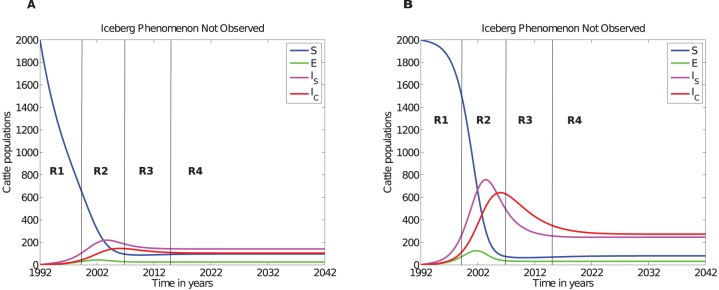
Predicted JD dynamics with estimated parameters . Predicted disease dynamics using parameters estimated through fitting model to prevalence with A) cattle life span of 5 years, and B) cattle life span of 20 years.

The ODE model was fitted to prevalence data. The data shows an increase in MAP prevalence over 3 years before a control program was implemented in 1994 and a decline in prevalence from 1994 to 1998 showing benefits of the control program. We fitted the model (*see* Figure 5) to the recorded prevalence before the control program was introduced (prevalence for years 1992 to 1994). This way of fitting gives an insight of the probable disease transmission rates and enables estimation of parameters associated with the observed prevalence. The estimated parameters (Table 4) were then used to simulate the disease dynamics (Figure 6) and to estimate animal ratios (Table 5) at each stage of the disease.

**Table 4:** Parameters estimated by fitting the ODE model to prevalence data.

Parameters Assuming 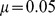	Estimated value RSS 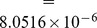	Parameters Assuming 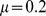	Estimated value RSS 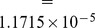
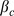	2.7035	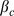	2.8428
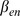	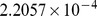	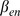	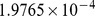

Only parameters that were estimated by fitting model to prevalence data are given. The rest of the parameters were fixed and are as given in Table 2. RSS means residual sum of squares. The least number of parameters to be fitted were determined using the collinearity or identifiability set of parameters method using the FME inverse modelling package [24], which enables detection of model parameters that are not correlated.

**Table 5:** Approximate ratios for animal populations in different stages of JD under different time regions (Figure 6 A).

Stage	R1	R2	R3	R4
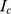 (Clinical)	100.0	400.0	350.0	250.0
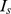 (Subclinical)	250.0	600.0	250.0	225.0
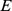 (Silent or exposed)	50.0	100.0	50.0	50.0
Ratio ( 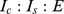 )	2:5:1	4:6:1	7:5:1	5:5:1

For ratios that correspond to Figure 6 B see file S2 in Supporting Information S1.

**Figure 7:**
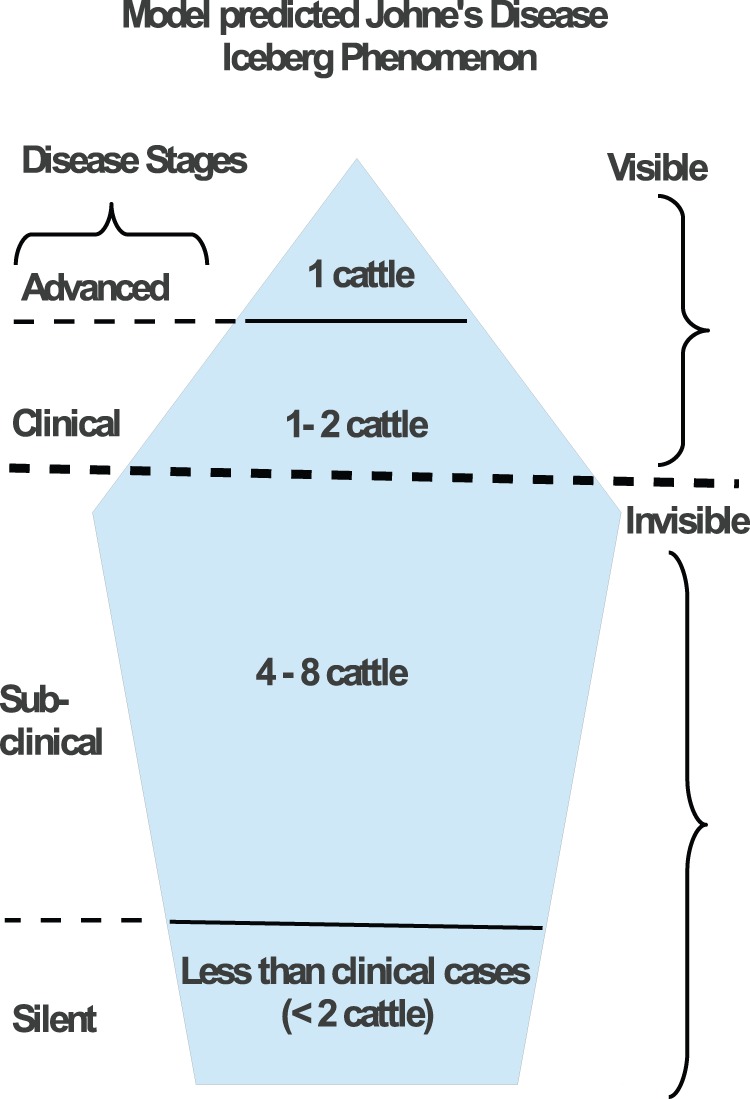
What the model predicts and suggests the iceberg phenomenon should be like in JD. Simulation results presented in Figures 2, 3, and 6, and Tables 3 and 5 demonstrate that there are always fewer cattle in the silent stage compared to the subclinical and clinical stages. We do not dispute that there are potentially more undiagnosed cases but suggests that the majority of these cases should be subclinical cases instead of cases in the silent stage.

Observe that the results presented in Figure 6 and Table 5 are qualitatively similar to those in Figure 2 and Table 3, respectively. See Figure 7 for an illustration of the iceberg phenomenon suggested by these results.

### Sensitivity analysis of ODE model results to parameters

**Figure 8:**
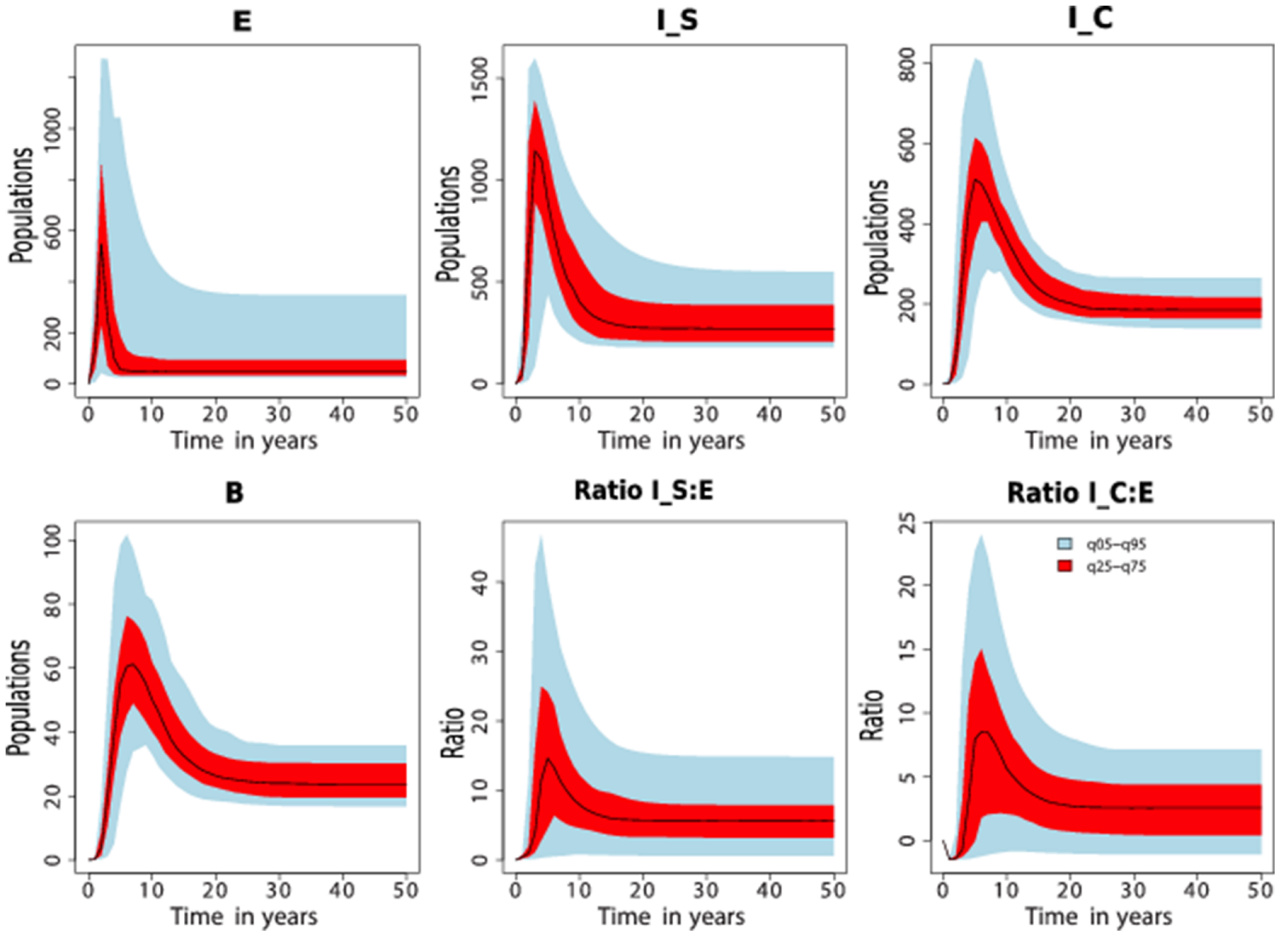
Sensitivity analysis. Sensitivity analysis of model output variables predicated that disease transmission dynamics when duration (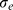, 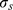, 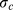), transmission (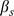, 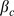, 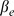) and environmental control (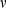) parameters are varied within ranges given in Table 5. The output variability shows high likelihood of environmental contamination whenever cattle are shedding MAP. Variation of 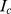 to 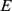 and 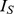 to 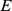 ratios are shown to be consistent with observations in Tables 3 and 5, and Figures 3 and 6.

Sensitivity analysis identifies parameters that are highly associated with disease transmission with a significant contribution to model output variability. This gives a measure to identify parameters that contribute more to rapid disease progression, hence providing insights to disease mechanisms that can be targeted for control. Sensitivity rankings of parameters given in Tables 6 and 7 suggest that disease transmission coming from the interaction of cattle with their environment is the main driving factor of disease transmission on a farm. This is followed in rank by 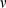, a parameter that models the bacilli life span in the environment. The longer the bacilli life span in the environment, the longer the environment will sustain disease persistence. The lowly ranked parameters are the transmission rates for the animals in the clinical and subclinical stages. The transmission rate of the clinical stage is ranked higher than the subclinical stage transmission rate. These transmission parameters are ranked lower than their associated duration periods. This suggests that the length of the duration periods influence the disease dynamics more significantly than the transmission parameters. Also, this result shows that MAP transmission is mainly driven by a contaminated environment, and animal-to-animal transmission is not the main driving factor of the disease. Figure 8 shows the associated output variable variability to multi-variations of these parameters.

**Table 6:** Sensitivity ranks of parameters used in the model in relation to predicated model outputs.

Parameter	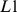	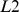	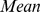	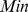	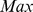
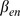	1.024325548	0.0689394301	0.510383674	−3.8687832682	3.7660858673
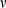	0.9510322297	0.0583787173	−0.537491563	−3.4010293696	3.023921857
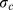	0.6526394453	0.0356476006	−0.4262092637	−1.7802387233	1.4477183877
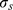	0.4600709931	0.0294405461	0.1616543217	−1.3202642822	1.8663175423
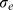	0.2610678851	0.0193499108	−0.1252005205	−1.1470696646	0.4714615448
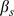	0.1294837836	0.0088125314	0.0614298468	−0.506887906	0.4981501685
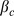	0.0735430833	0.0047273225	0.0369088358	−0.2485939043	0.2464713131

A: Univariate sensitivity indices of model parameters. Univariate sensitivity measures were calculated using FME inverse modelling package [24]. L1, L2, and Mean values rank the sensitivity of the parameters in the models. L1 = 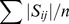, L2 = 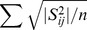, and 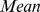, 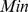 and 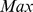 are the mean, min and max of the sensitivity functions (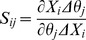, 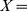 model variable and 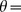 model parameter).

**Table 7:** Model parameter partial rank correlation coefficients (PRCCs) that cause significant model output variability.

( 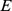 )		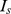		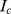		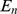	
Parameter	PRCCs	Parameter	PRCCs	Parameter	PRCCs	Parameter	PRCCs
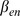	0.75715	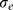	0.90842	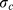	−0.85	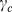	−0.91716
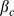	0.73791	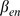	0.7001	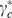	0.20891	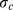	0.9118
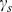	0.12511	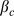	0.66705				

Given PRCC values are for the parameters found to be significant at 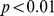 and 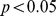, The 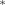 indicates that the associated parameter is not significant at 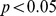. Only PRCC values at 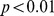 are given. Parameter ranges used are as given in Table 2.

### Effects of silent and subclinical time delays on disease dynamics: An alternative model

We developed and used a delay differential equation (DDE) model to further investigate the ratios of the number of animals in the silent (exposed), subclinical and clinical classes during the course of the disease. We use time delays to capture time cattle spend in the silent and subclinical stages before progressing to the next stages. Since there are no clearly defined boundaries between the silent and subclinical classes and between the subclinical class and clinical class, we introduce two time delays with parameters 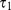 and 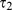, respectively, to cater for any additional time lapse at the borderlines of these two classes. This will ensure that cattle stay in their respective classes before entering subsequent classes and therefore the movement of animals in these classes will follow a gamma distribution than the exponential decay distribution assumed by the ODE model. This also allows for clear observation of the disease dynamics given disease stages with different duration periods, which define the disease dynamics. In this case, the cattle that advance to the clinical class on time 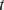 are cattle that were admitted into the exposed-subclinical borderline at an earlier time 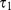, while the cattle that progress to the clinical stage are cattle that entered the subclinical-clinical stage borderline at an earlier time 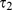, with 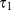 taking the range of 0 to 0.5 years and 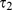 the range of 2 to 10 years. See file S3 in Supporting Information S1 for the system of equations of the DDE model (this model was only solved and analyzed numerically).

**Figure 9:**
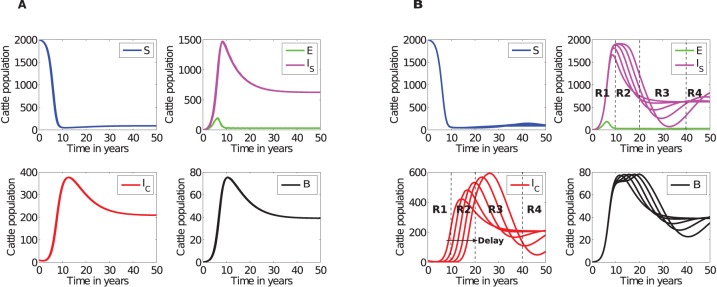
DDE model simulations. A) Varying the time delay 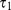 before exposed cattle (in the silent stage) move into the subclinical stage does not cause any significant change to the dynamics of the disease. B: Varying the delay 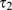 associated with time spent in the subclinical stage contributes to variable disease transmission dynamics and different levels of environmental contamination. With 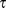 varied from 2 years to 10 years in steps of 2 years.

Comparing regions R1, R2, R3, and R4 in Figure 9 evinces that cattle in the subclinical stage are more than cattle in the clinical stage. There is no instance where cattle in the silent stage are more than cattle in subclinical and clinical stages. Simulations with delays clearly improve this picture by showing that the longer the subclinical incubation period the more time lapse before clinical cases are noticed. However, the silent stage delay has no significant influence on JD dynamics.

## Discussion and Conclusions

We developed a qualitative framework based on an ODE model to test the iceberg phenomenon and its application in JD to predict infection prevalence in a herd once a clinically infected animal is diagnosed. Prevalence data from Jubb and Galvin [22] was used to provide parameter estimates that were used to further test the model predictions. Numerical solutions of these models with the estimated parameters, as well as with parameters drawn from the JD literature, provide a qualitative picture of the disease transmission dynamics.

The first study [11] to predict the iceberg phenomenon and provide estimates for the ratios of the number of cattle at each stage of the disease (silent, subclinical, clinical and advanced) did not provide field data to substantiate the reported results. These ratios currently define the iceberg phenomenon in JD and have been cited in several studies [5], [11], [15], [16]. There are other longitudinal studies that were carried out to determine the prevalence of JD in farms in different regions [8], [19], [20], but none of these studies made an effort to prove the ratios used to describe the iceberg phenomenon in JD. According to our literature search, the study by Benedictus *et al*. [20] has the best observed field data of JD prevalence in a Pennsylvania dairy farm recorded over about 20 years. However, this study falls short to qualify to be used to validate the JD iceberg ratios because prevalence without control was recorded only for a period of about 1 year. In their study, Jubb and Galvin [22], implemented a control program after recording number of clinical cases for 3 years. Modelling of the control program in the study of Jubb and Galvin [22] resulted in the estimation of model parameters that lead to the control of the disease (results not shown), which does not aid in getting a better understanding of the JD iceberg ratios. Even though we used the prevalence data from that study [22] before the control was introduced, it does not provide a clear representation of the natural course of JD, which has a long subclinical duration period of 2 to 10 years. It is recommended to put into place measures that control disease transmission in farms. Implementation of disease control programs in those studies [20], [22] confounded information and makes it difficult to estimate true disease prevalence in the absence of control programs. This is where we think our modeling becomes relevant and can provide predictions that explain what those studies cannot show. Results predicted in this study revealed that the ratios reported in previous studies to be associated with JD require further investigation (*see* Figures 6 and 7 and Table 5). This will be a difficult field study to carry out because of the slow progressive nature of the disease and the associated limited methods to diagnose the number of cattle in each class at different disease stages since it is difficult to correctly detect silent and subclinical cases.

Our models demonstrate that the number of subclinical shedding animals is higher than animals in the silent or exposed class, which is different from what is reported in previous studies. Model predictions based both on generally acceptable parameters and parameters estimated through model fitting to prevalence data recorded in the Judd and Galvin [22] study show that it is impossible to observe the JD iceberg ratios when disease duration periods that are associated with the natural course of the disease progression are used. Our modelling procedure does not estimate exact ratios but approximate ratios that are qualitatively similar in the spectrum of the duration periods associated with the natural course of the disease. However, the ratios that describe the current JD iceberg phenomenon can be illustrated under unrealistic assumptions. These assumptions require that the silent stage duration period be greater than that of the subclinical stage (which is known to span from 2 to 10 years). The model results suggest that the JD iceberg ratios falls short in estimating correctly the number of cattle that should be in the silent stage when a clinical case is detected. The model predicts that in different time regions R1, R2, R3 and R4 (*see* Figures 2, 3 and 6, and Tables 3 and 5) there are approximate ratios of clinical cattle: subclinical cattle: exposed cattle of about 1∶4∶1, 3∶5∶1, 10∶6∶1, and 5∶5∶1, respectively. These ratios qualitatively agree with the current JD iceberg ratios in estimating the clinical and the subclinical stage cases, but do not agree with the prediction of number of animals that should be in the silent stage or exposed animals. We further tested these results with a DDE model with two time delays, (i) the first delay in the silent stage before progressing into the subclinical stage and (ii) the second delay in the subclinical stage before animals progress into the clinical stage. The DDE model paints a clearer picture of these ratios (*see* Figure 9), the longer the time animals spend in the subclinical class, the more the animals will accumulate in this class. The reserve is not true for the exposed (or silent class) because of a shorter residence time interval. The observed ratios will not change even if the residence time for the exposed class is increased to 2 years. Any residence time for the exposed class that is less than that of the subclinical class will not yield the JD iceberg. One possible explanation is that, in the previous reports, most of animals categorised in the exposed class might indeed be subclinical and misdiagnosed by the fecal culture tests, since current fecal culture tests can miss intermittent and very low shedders.

Models can play an important role in explaining disease transmission and persistence mechanisms. They have the potential to predict testable hypotheses and explain what is not clearly known or difficult to test or detect experimentally. Our analysis identified parameters and threshold parameter groupings, such as the basic reproduction number, that are critical in the control of JD. For example, the numerator of the basic reproduction number contains additive terms in the three transmission rates 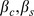, and 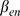. This poses a major problem in disease control as it indicates that control measures should be thought of as well as designed to target all three major ways of disease transmission. Note that from the form of 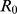, if the environment is cleaned such that cattle-environment-cattle transmission is not possible, but subclinical and clinical cattle are not treated, the disease can still persist or establish itself within a farm. On the other hand, if control measures target only subclinical and clinical cattle but not environmental cleaning, the disease can still establish itself in the farm. Thus, our model explains why it may be difficult to eradicate JD. More importantly, our model predicts that whenever there is a clinical case detected there are not as many animals in the silent stage as in the subclinical or clinical phases of the disease as was thought or reported. The results also demonstrate that the best way to prevent infection persistence, transmission and spread of JD is by reducing infection exposure and maintaining a bacilli-free environment. A contaminated environment appears to be a stronger driver of infection persistence and transmission compared to transmissions associated with animal-to-animal interactions. These predictions are based on a model that did not separate cattle into different age groups. Our future focus will be to develop and explore an age-structured model that addresses the same questions.

In conclusion, as developing early diagnostic and detection procedures will be beneficial to controlling the disease, preventive vaccines, like Bacille Calmette Guerin in human tuberculosis, may be the best in preventing JD. Such vaccines, if developed, will especially prevent calves from getting infected when exposed. If coupled with good environmental hygiene practices, this could be pivotal in eradicating the disease.

## Supporting Information

Figure S1
**Simulated JD farm dynamics and ratios**: An illustration of MAP transmission in a farm with animal removal rate that corresponds to farm animal life span of 5 years (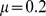.)(EPS)

Supporting Information S1 **Contains:** Suppplementary File S1, File S2 and File S3.
(PDF)
