# Correction: Evaluation of the “Iceberg Phenomenon” in Johne's Disease through Mathematical Modelling

**DOI:** 10.1371/annotation/44f299df-fbe6-4ed2-b802-1616e2cb36ee

**Published:** 2013-11-07

**Authors:** Gesham Magombedze, Calistus N. Ngonghala, Cristina Lanzas

The name of the first author was spelled incorrectly. The correct name is: Gesham Magombedze.

In addition, the word "Evaluation" was spelled incorrectly in the article title. The correct title is: Evaluation of the "Iceberg Phenomenon" in Johne's Disease through Mathematical Modelling.

The correct citation is: Magombedze G, Ngonghala CN, Lanzas C (2013) Evaluation of the “Iceberg Phenomenon” in Johne's Disease through Mathematical Modelling. PLoS ONE 8(10): e76636. doi:10.1371/journal.pone.0076636.

In addition, the number of the grant from the National Science Foundation is incorrect. The correct grant number is: NSF Award # EF-0832858. 

