# Supplementary material for: Evalution of the “Iceberg Phenomenon” in Johne's Disease through Mathematical Modelling
Source: PLoS One. 2013 Oct 22;8(10):e76636. doi: 10.1371/journal.pone.0076636 (PMC3805542; doi:10.1371/journal.pone.0076636)
Supplement: Supporting Information S1 — Contains: Suppplementary File S1, File S2 and File S3. (PDF) [file pone.0076636.s002.pdf]

Supplementary file

Model analysis: Supplementary file S1

Positivity and boundedness of solutions

We show that the model system defined by the system of equations

$$\begin{aligned}
\frac{dS(t)}{dt} &= \Lambda - \left(\frac{\beta_s I_s(t) + \beta_c I_c(t)}{N} + \beta_{en} E_n(t)\right) S(t) - \mu S(t), \\
\frac{dE(t)}{dt} &= \left(\frac{\beta_s I_s(t) + \beta_c I_c(t)}{N} + \beta_{en} E_n(t)\right) S(t) - (\sigma_e + \mu) E(t), \\
\frac{dI_s(t)}{dt} &= \sigma_e E(t) - (\sigma_s + \mu) I_s(t), \\
\frac{dI_c(t)}{dt} &= \sigma_s I_s(t) - (\sigma_c + \mu) I_c(t),
\end{aligned} \tag{S.1}$$

where

$$\frac{dN(t)}{dt} = \Lambda - \mu N(t) - \sigma_c I_c(t) \tag{S.2}$$

is well-posed from a mathematical and biological stand-point and that solutions to the system are always positive and bounded within the region defined by

$$\Phi \subset \mathbb{R}^4_+, \text{ where } \Phi = \left\{ (S, E, I_s, I_c) \in \mathbb{R}^4_+ : 0 \leq S + E + I_s + I_c = N \leq \frac{\Lambda}{\mu} \right\}.$$

First, we note that all model parameters and variables are non-negative. If initial conditions of the form $(S(0), E(0), I_s(0), I_c(0)) = (S^0, E^0, I_s^0, I_c^0,)$ for system (S.1) are provided in Φ, then using standard techniques from [1, 2], we can show that this system has a unique solution in Φ, which depends continuously on the initial conditions.

The dynamics of the total cattle population is governed by the equation

$$\begin{aligned}
\frac{dN(t)}{dt} &= \Lambda - \mu N(t) - \sigma_c I_c(t), \\
&\leq \Lambda - \mu N(t).
\end{aligned}$$

Therefore,

$$\begin{aligned}
N(t) &\leq \frac{\Lambda}{\mu} + \left(N(0) - \frac{\Lambda}{\mu}\right) e^{-\mu t}, \\
&\leq \frac{\Lambda}{\mu} \text{ if } N(0) \leq \frac{\Lambda}{\mu},
\end{aligned}$$

where $N(0) = N^0$ is the initial size of the total cattle population. Thus, Φ is positively-invariant and attracting with respect to system (S.1), and so the system is well-posed in Φ.

Basic reproduction number

We use the next generation matrix method [3, 4] to compute the basic reproduction number of our model. By this method, the basic reproduction number of our model is the spectral radius of the next generation matrix

$$\mathscr{F}\mathscr{V}^{-1} = \begin{pmatrix}
\frac{\beta_s\sigma_e}{B_1B_2} + \frac{\beta_c\sigma_e\,\sigma_s}{B_1B_2B_3} + \frac{\beta_e\Lambda\,\sigma_e\,(\alpha_s B_3 + \alpha_c\sigma_s)}{\mu\, B_1B_2B_3\nu} & \frac{\beta_s}{B_2} + \frac{\beta_c\sigma_s}{B_2B_3} + \frac{\beta_e\Lambda\,(\alpha_s B_3 + \alpha_c\sigma_s)}{\mu\, B_2B_3\nu} & \frac{\beta_c}{B_3} + \frac{\beta_e\Lambda\,\alpha_c}{\mu\, B_3\nu} & \frac{\beta_e\Lambda}{\mu\,\nu} \\
0 & 0 & 0 & 0 \\
0 & 0 & 0 & 0 \\
0 & 0 & 0 & 0
\end{pmatrix},$$

where

$$
\begin{aligned}
B_1 &= \sigma_e + \mu, B_2 = \sigma_s + \mu, B_3 = \gamma + \mu, \\
\mathscr{F} &= \begin{pmatrix} 0 & \beta_s & \beta_c & \frac{\beta_{en}\Lambda}{\mu} \\ 0 & 0 & 0 & 0 \\ 0 & 0 & 0 & 0 \\ 0 & 0 & 0 & 0 \end{pmatrix}, \mathscr{V} = \left[(\begin{matrix} \sigma_e + \mu & 0 & 0 & 0 \\ -\sigma_e & \sigma_s + \mu & 0 & 0 \\ 0 & -\sigma_s & \gamma + \mu & 0 \\ 0 & -\alpha_s & -\alpha_c & \nu \end{matrix} \right) .
\end{aligned}
$$

That is,

$$
\begin{aligned}
\mathscr{R}_0 = \rho(\mathscr{F}\mathscr{V}^{-1}) &= \frac{\sigma_e\,(\beta_s \mu\, B_3 \nu + \beta_c \sigma_s\, \mu\, \nu + \beta_e \Lambda\, \alpha_s B_3 + \beta_e \Lambda\, \alpha_c \sigma_s)}{\mu\, B_1 B_2 B_3 \nu} \\
&= \frac{\sigma_e(\beta_{en}\Lambda(\gamma_s(\gamma+\mu) + \gamma_c \sigma_s) + \mu\nu(\beta_s(\gamma+\mu) + \beta_c \sigma_s))}{\mu\nu(\sigma_e+\mu)(\sigma_s+\mu)(\gamma+\mu)}.
\end{aligned}
$$

Note that the matrix $\mathscr{F}$ is made up of new infections, while the matrix $\mathscr{V}$ consists of terms depicting the transfer of infection from one class to another. Also, observe that the value of the basic reproduction obtained through the next generation operator approach is the same as that obtained by seeking conditions under which a transcritical bifurcation exists.

Existence and stability of equilibrium solutions

The equilibrium solutions of system (S.1) together with the equation

$$
\frac{dE_n(t)}{dt} = \gamma_s I_s(t) + \gamma_c I_c(t) - \nu E_n(t), \tag{S.3}
$$

are obtained by setting the right hand sides of the equations to zero and solving the resulting system of algebraic equations for the variables. This process indicates that equations (S.1) and (S.3) has a disease-free equilibrium solution $\mathcal{E}_0 = \left(\frac{\Lambda}{\mu}, 0, 0, 0, \frac{\Lambda}{\mu}, 0\right)$ when $R_0 \leq 1$ and a unique stable endemic equilibrium solution $\mathcal{E}_e = (S^*, E^*, I_s^*, I_c^*, N^*, E_n^*)$, which can be expressed in terms of I_c^*, the endemic equilibrium value of the clinically sick cattle as follows:

$$
\begin{aligned}
S^* &= \frac{\Lambda\sigma_e\sigma_s\nu(\Lambda - \sigma_c I_c^*)}{\Lambda\mu\nu\sigma_e\sigma_s + (\mu\nu(\sigma_e+\mu)(\sigma_s+\mu)(\gamma+\mu)R_0 - \sigma_c\sigma_e(\beta_{en}(\gamma_s(\sigma_c+\mu) + \sigma_s\gamma_c)I_c^* + \mu\sigma_s\nu))I_c^*}, \\
E^* &= \frac{(\sigma_s+\mu)(\sigma_c+\mu)I_c^*}{\sigma_s\sigma_e}, \quad I_s^* = \frac{(\sigma_c+\mu)I_c^*}{\sigma_s}, \quad N^* = \frac{\Lambda - \sigma_c I_c^*}{\mu}, \quad E_n^* = \frac{(\gamma_s(\sigma_c+\mu) + \gamma_c\sigma_s)I_c^*}{\sigma_s\nu},
\end{aligned}
$$

with I_c^* given by the equation

$$
(I_c^{*2} + a_1 I_c^* + a_0) = 0, \tag{S.4}
$$

where

$$
\begin{aligned}
a_1 &= -\frac{\mu\nu\sigma_s}{\beta_{en}(\gamma_s(\sigma_c+\mu) + \gamma_c\sigma_s)}\left(\frac{\Lambda\sigma_e\beta_{en}(\gamma_s(\sigma_c+\mu) + \gamma_c\sigma_s)}{\mu\nu(\sigma_e+\mu)(\sigma_s+\mu)(\sigma_c+\mu)} + \frac{(\sigma_e+\mu)(\sigma_s+\mu)(\sigma_c+\mu)R_0}{\sigma_e\sigma_s\sigma_c} - 1\right), \\
a_0 &= \frac{\Lambda\mu\nu\sigma_e\sigma_s(R_0 - 1)}{\beta_{en}\sigma_c(\gamma_s(\sigma_c+\mu) + \gamma_c\sigma_s)}.
\end{aligned}
$$

Equation (S.4) indicates that $\mathcal{E}_0$, always exists, and that when $R_0 > 1$, we can have one epidemiologically feasible endemic equilibrium solution. This endemic equilibrium solution can be computed in closed form by solving the quadratic portion of equation (S.4) in $\mathbb{R}_+$. The existence of an endemic equilibrium solution depicts persistence of JD within a cattle population.

Theorem 1. *The DFE of the system given by equations* (S.1) *is both locally and globally asymptotically stable when* $\mathscr{R}_0 < 1$. *When* $\mathscr{R}_0 > 1$, $\mathcal{E}_0$ *is unstable and there exists an endemic equilibrium solution* $\mathcal{E}_e$

Proof. We establish the proof of Theorem 1. From the calculations leading to the basic reproduction number, it is clear that the disease-free equilibrium is locally and asymptotically stable when $\mathscr{R}_0 < 1$.

We now use the next generation matrix approach to establish the global stability of the disease-free steady state. To achieve this, we set $X = S$, $Z = (E, I_s, I_c, E_n)$ and split equations (S.1)-(S.3) into two subsystems – an equation in X, or a disease-free equation and a collection of four equations in Z, consisting of the second, third and fourth equations of system (S.1) and Equation (S.3) as follows:

$$\begin{aligned} \dot{X} &= F(X, 0), \\ \dot{Z} &= G(X, Z). \end{aligned}$$

Clearly, the disease-free steady state $\mathcal{E}_0$ is locally and asymptotically stable when $\mathscr{R}_0 < 1$ and the only equilibrium solution $(X^*, 0)$, of the disease-free equation is globally and asymptotically stable. Hence, by the approach in [5] , all we need to do in order to establish the global stability of the disease-free equilibrium is to show that $\hat{G}(X, Z) = D_Z(X^*, 0) - G(X, Z) \geq 0$ in Φ, where $D_Z(X^*, 0) - G(X, Z)$ is the Jacobian of G evaluated at the disease-free equilibrium $(X^*, 0)$. But

$$\hat{G}(X, Z) = \begin{pmatrix} (\beta_s I_s + \beta_c I_c)\left(1 - \frac{S}{N}\right) + \beta_{en} E_n \left(\frac{\Lambda}{\mu} - S\right) \\ 0 \\ 0 \\ 0 \end{pmatrix}.$$

Since $0 < S \leq N \leq \frac{\Lambda}{\mu}$, $(\beta_s I_s + \beta_c I_c)\left(1 - \frac{S}{N}\right) + \beta_{en} E_n \left(\frac{\Lambda}{\mu} - S\right) \geq 0$. Clearly, $\hat{G}(X, Z) \geq 0$. Thus, the disease-free equilibrium is globally and asymptotically stable.

Special case, $\sigma_c = 0$

Assuming that JD does not kill cattle, the basic reproduction number for the system reduces to

$$\mathscr{R}_0 = \frac{\sigma_e(\beta_{en}\Lambda(\gamma_s\mu + \gamma_c\sigma_s) + \mu\nu(\beta_s\mu + \beta_c\sigma_s))}{\mu^2\nu(\sigma_e + \mu)(\sigma_s + \mu)}, \tag{S.5}$$

and the equilibrium solutions now become $\mathcal{E}_0 = \left(\frac{\Lambda}{\mu}, 0, 0, 0, \frac{\Lambda}{\mu}, 0\right)$ and $\mathcal{E}_e = (S^*, E^*, I_s^*, I_c^*, N^*, E_n^*)$, where

$$\begin{aligned} S^* &= \frac{\Lambda^2\sigma_s\nu\mu\sigma_e(\sigma_e + \mu)(\sigma_s + \mu)\mathscr{R}_0}{(\mu\nu(\beta_s\mu + \beta_c\sigma_s) + \beta_{en}(\gamma_s\mu + \gamma_c\sigma_s)\Lambda)\Lambda\sigma_s(\mathscr{R}_0 - 1) + \sigma_s\mu\nu\Lambda\mu\sigma_e(\sigma_e + \mu)(\sigma_s + \mu)\mathscr{R}_0}, \\ E^* &= \frac{(\sigma_s + \mu)\mu}{\sigma_s\sigma_e}\frac{\Lambda\sigma_s(\mathscr{R}_0 - 1)}{\mu\sigma_e(\sigma_e + \mu)(\sigma_s + \mu)\mathscr{R}_0}, \quad I_s^* = \frac{\mu}{\sigma_s}\frac{\Lambda\sigma_s(\mathscr{R}_0 - 1)}{\mu\sigma_e(\sigma_e + \mu)(\sigma_s + \mu)\mathscr{R}_0}, \\ I_c^* &= \frac{\Lambda\sigma_s(\mathscr{R}_0 - 1)}{\mu\sigma_e(\sigma_e + \mu)(\sigma_s + \mu)\mathscr{R}_0}, \quad E_n^* = \frac{\gamma_s\mu + \gamma_c\sigma_s}{\sigma_s\nu}\frac{\Lambda\sigma_s(\mathscr{R}_0 - 1)}{\mu\sigma_e(\sigma_e + \mu)(\sigma_s + \mu)\mathscr{R}_0}, \quad N^* = \frac{\Lambda}{\mu}. \end{aligned}$$

This indicates that the endemic equilibrium $\mathcal{E}_e$ only exists when $\mathscr{R}_0 > 1$. Note that $\mathcal{E}_e$ simplifies to $\mathcal{E}_0$ when $\mathscr{R}_0 \leq 1$. Following standard nonlinear dynamics techniques it can easily be shown that the $\mathcal{E}_0$ is globally and asymptotically stable when $\mathscr{R}_0 < 1$ and unstable when $\mathscr{R}_0 > 1$. It can also be shown that $\mathcal{E}_e$ is stable when $\mathscr{R}_0 > 1$.

Additional results: Supplementary file S2

See Figure S1, and Tables S1 and S2 for additional farm ($\mu = 0.2$) JD predicted dynamics and ratios.

Delay differential equation (DDE) model: Supplementary file S3

The transmission model is modified into an DDE model and the equations for $E(t)$, $I_s(t)$, and $I_c(t)$ take the forms:

$$\begin{aligned}\frac{dE(t)}{dt} &= \left(\frac{\beta_s I_s(t)+\beta_c I_c(t)}{N(t)} + \beta_{en}E_n(t)\right)S(t) - \sigma_e E(t-\tau_1) - \mu E(t),\\ \frac{dI_s(t)}{dt} &= \sigma_e E(t-\tau_1) - \sigma_s Is(t-\tau_2) + \mu I_s(t),\\ \frac{dI_c(t)}{dt} &= \sigma_s I_s(t-\tau_2) - (\sigma_c+\mu)I_c(t).\end{aligned} \tag{S.6}$$

See Table S3 for the parameters of the DDE model.

References

[1] Hale HK (1969) Ordinary differential equations. New York: John Wiley & Sons, 296–297 pp.

[2] Jordan DW, Smith P (1999) Nonlinear Ordinary Differential Equations. An Introduction to Dynamical Systems. oxford, UK: Oxford University Press, 3rd edition.

[3] Diekmann O, Heesterbeek JAP, Metz JAJ (1990) On the definition and the computation of the basic reproduction ratio R_0 in models for infectious diseases in heterogeneous populations. J Math Biol 28: 365–382.

[4] van den Driessche P, Watmough J (2002) Reproduction numbers and sub-threshold endemic equilibria for compartmental models of disease transmission. Math Biosci 180: 29–48.

[5] Castillo-Chavez C, Blower S, van den Driessche P, Kirschner D, Yakubu AA (2002) Mathematical approaches for emerging and reemerging infectious diseases. New York: Springer-Verlag.

Supplementary tables

Table S1: **Ratios for animal populations in different stages of JD under different time regions**. Approximate ratios of animals in the Silent, Subclinical, and Clinical stages at a particular time in a given region. These ratios show that the JD Iceberg phenomenon cannot be observed.

Approximate cattle population at each time point in Region	R1	R2	R3	R4
I_c	25.0	100.0	125.0	100.0
I_s	50.0	200.0	150.0	150.0
E	25.0	50.0	25.0	25.0
Ratio ($I_c : I_s : E$)	1:2:1	2:4:1	5:6:1	4:6:1

Table S2: **Ratios for animal populations in different stages of JD under different time regions using estimated parameters**. Approximate ratios of animals in the Silent, Subclinical, and Clinical stages at a particular time in a given region with $\mu = 0.2$. These ratios show that the JD Iceberg phenomenon cannot be observed.

Approximate cattle population at each time point in Region	R1	R2	R3	R4
I_c	25.0	50.0	50.0	50.0
I_s	50.0	100.0	75.0	75.0
E	25.0	25.0	25.0	25.0
Ratio ($I_c : I_s : E$)	1:2:1	2:4:1	2:3:1	2:3:1

Table S3: **Time delays in the silent and subclinical stages**.

Time delay	Stage of infection	Delay interval
τ_1	Silent stage delay	0-0.5 years
τ_2	Subclinical stage delay	2-10 years
